# Bilateral Stress Fractures of the Femoral Neck after Total Knee Arthroplasty: Importance of Early Diagnosis

**DOI:** 10.1155/2020/3091693

**Published:** 2020-02-06

**Authors:** Sei Morinaga, Kenichi Ueshima, Yoshinobu Maruhashi, Noriyuki Hashimoto, Shoji Watanabe, Takuya Nakamura

**Affiliations:** ^1^Department of Orthopedics Surgery, Toyama Prefectural Central Hospital, Japan; ^2^Department of Orthopaedic Surgery, Graduate School of Medical Science, Kanazawa University, Kanazawa, Japan; ^3^Watanabeseikeigekaiin, Japan

## Abstract

Unilateral stress fracture of the femoral neck following total knee arthroplasty (TKA) is a rare complication; only 21 cases are described in English literature so far. Bilateral stress fractures of the femoral neck occurring simultaneously following a bilateral TKA have been seen in only 2 cases till now. We report a patient suffering from rheumatoid arthritis of both knees, who was treated with bilateral TKA. She developed spontaneous fractures of the femoral neck on both sides 12 months following the TKA. She was treated with bilateral total hip arthroplasty (THA). Stress fracture of the femoral neck should be suspected in patients complaining of hip pain who have undergone TKA.

## 1. Introduction

Stress fractures of the femoral neck have been described either as a result of excessive loading of the hip in a patient with normal bones such as athletes and military recruits, or as a result of normal loading of the hip in a patient with weakened bones, such as those with osteoporosis and rheumatoid disease who are receiving steroid therapy [1]. Unilateral stress fractures of the femoral neck following total knee arthroplasty (TKA) are a rare complication; only 21 cases have been described so far [2–10]. To the best of our knowledge, there are only two reported cases of bilateral stress fractures of the femoral neck occurring simultaneously following a bilateral TKA [11, 12]. We now report a third case of bilateral stress fractures of the femoral neck occurring simultaneously following a bilateral TKA.

## 2. Case Report

**Figure 1:**
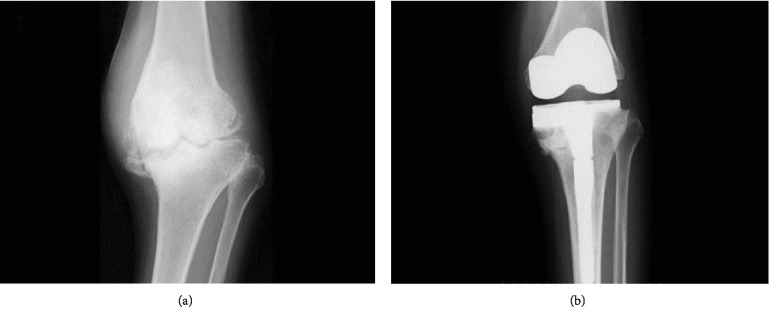
(a) Preoperative radiograph of the left knee showing severe varus. (b) Postoperative radiograph after left TKA.

A 57-year-old woman with a weight of 52 kg, a BMI of 20.3, and a 6-year history of rheumatoid arthritis in both knees was referred to the authors for TKA. She had severe pain in both knees, required a cane while walking, and could walk only a short distance. She was taking methotrexate 8 mg per week, methylprednisolone 3 mg per day, and bucillamine 200 mg per day. Dual-energy X-ray absorptiometry of the left radius was 0.674 g/cm^2^ and T score was 104%. On examination, the right femorotibial angle was 180° and the left was 198°. There was significant destruction of the left knee bone. The flexion angles were 90° on the right side and 105° on the left side, while extension angles were -10° and -20° on the right and left sides, respectively. There were no other medical comorbidities. She was treated with left TKA in December 2015 (Persona PS Femoral, Persona PS Surface, NexGen Stemed Tibia Stem Extension, and Tibia Augment 10 mm Half Block, Zimmer) (Figure 1). We decreased biomechanical loading due to excessive correction and maintained adequate balancing of the ligaments. Rehabilitation was started 24 hours after surgery without any weight restrictions. Postoperatively, recovery was uneventful and she was discharged walking with a cane.

**Figure 2:**
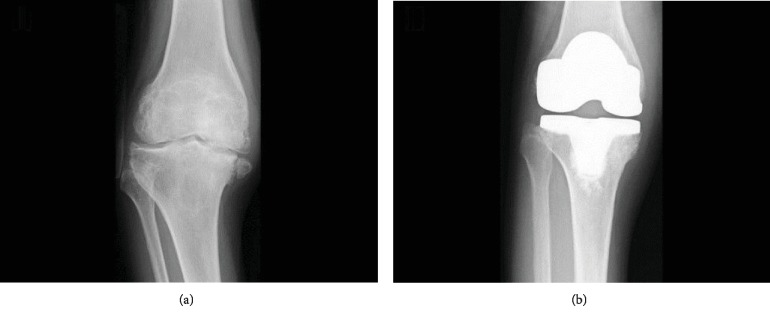
(a) Preoperative radiograph of the right knee showing mild varus. (b) Postoperative radiograph after right TKA.

She underwent right TKA in January 2016 (Persona PS Femoral, Persona PS Surface, and Persona Stemmed Tibia, Zimmer) (Figure 2). We decreased biomechanical loading due to excessive correction and maintained adequate balancing of the ligaments like the previous surgery. Rehabilitation was started 24 hours after surgery without any weight restrictions. On discharge, she could walk without a cane.

**Figure 3:**
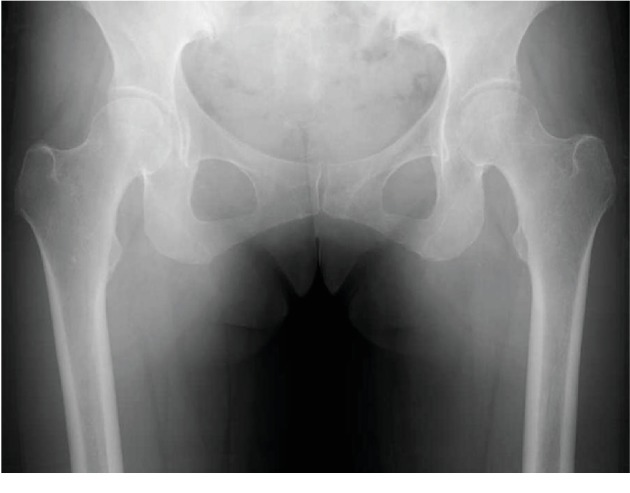
Radiograph of the hip 10 months after TKA.

In November 2016, 10 months after TKA, she developed bilateral groin pain without any trauma; therefore, she visited a nearby hospital. The initial radiograph did not indicate a fracture (Figure 3). Rheumatoid factor was negative.

**Figure 4:**
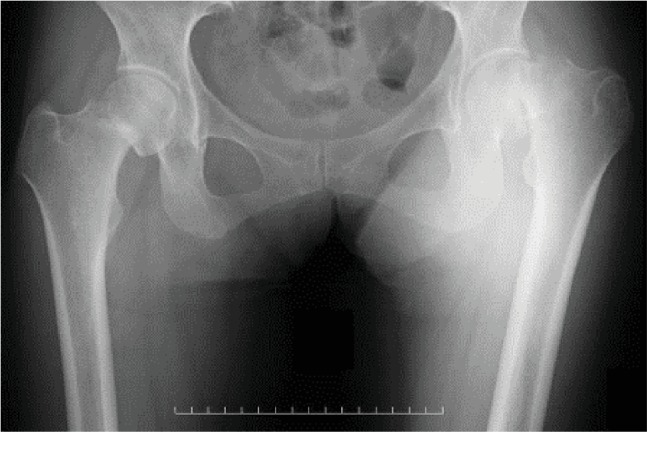
Radiograph of the hip 12 months after TKA.

She was treated with rest and anti-inflammatory drugs. Her pain continued, and 2 months later, she was unable to walk without support. Radiographs of the hip showed displaced subcapital fractures of the femoral neck on both sides (Figure 4).

**Figure 5:**
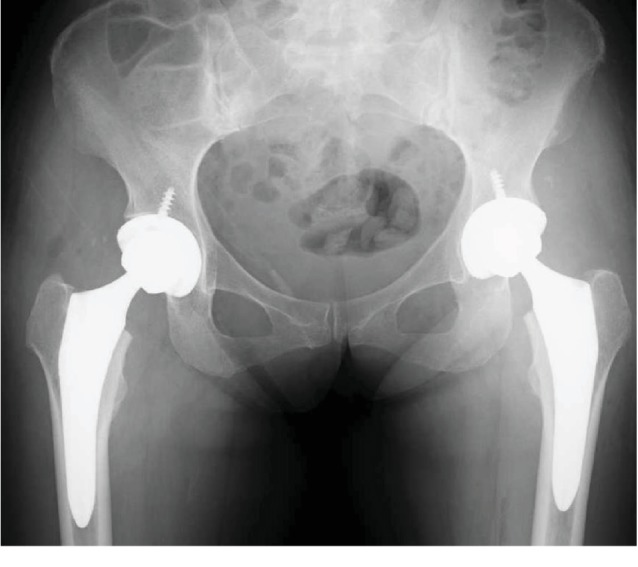
Postoperative radiograph of the bilateral THA.

She was treated with bilateral THA (SQRUM HA Cup, AQUALA, J-taper Stem, AZUL Head 32 mm ± 0, Kyocera) (Figure 5). The immediate postoperative period was uneventful, and she was allowed to bear full weight and walk with a walker. She was discharged with a cane. Six months after the operation, she was pain free and walked without any support. She takes sodium risedronate hydrate 17.5 mg per week for osteoporosis.

## 3. Discussion

Unilateral stress fracture of the femoral neck following TKA is a rare complication; only 21 cases have been described in English literature so far [2–10] (Table 1). There are only two cases of bilateral stress fractures of the femoral neck occurring simultaneously following a bilateral TKA [11, 12]. The most common risk factors for these fractures are an increased level of activity of the patient after TKA [2–10, 12], osteoporosis [2, 6–8, 10, 12], rheumatoid arthritis [7], steroid intake [7], preoperative valgus deformity of the knee and altered biomechanics following TKA [5, 9], and insertion of a rotating hinge prosthesis [5, 7, 11]. We think that the causes of fracture in our patient were rheumatoid arthritis, steroid intake, osteoporosis, and increased activity level after TKA. Joshi et al. [9] stated that changes in the biomechanical axis of the hip resulting from the correction of valgus knees create a situation in which increased stresses on the femoral neck potentiate the risk of stress fracture. Out of 24 cases (varus in our case), valgus deformity was described only in five. We should suspect a femoral neck stress fracture in patients complaining of hip pain who have undergone TKA with both varus and valgus deformity. Biomechanical studies are needed to evaluate the knee and hip kinematics after TKA [11]. The interval between TKA and the development of a stress fracture of the femoral neck has been described from 2 to 33 months, with an average of 8.7 months (12 months in our case). Diagnosing stress fractures based on radiographs is difficult. Some studies have described that a bone scintigram helps diagnosis [2, 5]. Hendel et al. reported that a bone scintigram was negative for a femoral neck stress fracture following total knee arthroplasty [8]. Several reports state that femoral neck fractures were diagnosed based on magnetic resonance imaging (MRI) after the bone scan was negative [13, 14]. Additional disadvantages of bone scintigrams are the long time required for the results, high radiation doses to patients, and high costs.

MRI is minimally invasive, safer, and provides a diagnosis earlier. Timely diagnosis is very important as internal fixation of the fracture may prevent displaced fractures, thus preserving the patient's own hip joint and avoiding arthroplasty [3, 9]. If an MRI has been done when the patient applied with hip pain, with early diagnosis the fracture should be prevented by prophylactic nailing or some careful other treatments. Clinicians should suspect stress fractures of the femoral neck in patients with hip pain following TKA and take MRI.

## Figures and Tables

**Table 1:** Comprehensive data of published literature.

	No.	Unilateral or bilateral (fracture)	Deformity (knee)	Interval (months)
Lesniewski and Testa [2]	1	Unilateral	NA	3
McElwaine and Sheehan [3]	7	Unilateral	Valgus: 1 Varus: 6	3-16
Fipp [4]	2	Unilateral	Varus	6, 7
Hardy et al. [5]	1	Unilateral	NA	12
Palanca et al. [6]	1	Unilateral	NA	17
Rawes et al. [7]	3	Unilateral	Varus: 2 NA: 1	2, 3, and 5
Hendel et al. [8]	1	Unilateral	NA	6
Joshi et al. [9]	4	Unilateral	Valgus: 4	6-13
Atalar et al. [10]	1	Unilateral	NA	NA
Pankaj et al. [11]	1	Bilateral	Both varus	9
Cakmak et al. [12]	1	Bilateral	Both varus	33
Current report	1	Bilateral	Both varus	12

NA: information not available.
